# Leadership and Burnout in Anatomic Pathology Laboratories: Findings from Greece’s Attica Region

**DOI:** 10.3390/healthcare14010077

**Published:** 2025-12-27

**Authors:** Angeliki Flokou, Sofia Pappa, Vassilis Aletras, Dimitris A. Niakas

**Affiliations:** 1 School of Social Sciences, Hellenic Open University, 26331 Patra, Greece; dimitris.niakas@gmail.com; 2 Medical School, National and Kapodistrian University of Athens, 11527 Athens, Greece; 3 Sismanogleio General Hospital, 15126 Athens, Greece; sofia.pappa.83@gmail.com; 4 Department of Business Administration, School of Business Administration, University of Macedonia, 54636 Thessaloniki, Greece; valetras@uom.edu.gr

**Keywords:** occupational burnout, anatomic pathology, leadership styles, transformational leadership, management by exception (passive), Copenhagen Burnout Inventory (CBI), Multifactor Leadership Questionnaire (MLQ-5X)

## Abstract

Background: Anatomic pathology laboratories operate under conditions requiring high precision, strict documentation, biosafety protocols, and tight turnaround times. Evidence from Greece is limited, and joint assessment of burnout and leadership in this setting is rare. Objective: The aim of this study was to estimate burnout levels among anatomic pathology personnel in Attica and examine their association with perceived leadership style. Methods: A cross-sectional survey of public and private laboratories was carried out. The questionnaire included demographics and work characteristics, the Copenhagen Burnout Inventory (CBI), and the Multifactor Leadership Questionnaire Form 5X (MLQ-5X). Results: Burnout levels were moderate to low overall, with personal burnout highest, work-related intermediate, and colleague-related lowest. Women and employment type were associated with personal burnout (*p* < 0.05). Passive/avoidant leadership (including management by exception–passive and laissez-faire) showed positive associations with burnout, whereas transformational leadership and favorable leadership outcomes—particularly, perceived effectiveness and satisfaction with the leader—were inversely associated; transactional leadership followed the same direction but less robustly (*p* < 0.05 where supported). Conclusions: Burnout among anatomic pathology personnel in Attica is non-trivial and varies across domains. Leadership dimensions display differential links with burnout, indicating potentially modifiable organizational targets for intervention. Significance: To our knowledge, this is the first study in Greece and among the first in Europe to jointly apply CBI and MLQ-5X in anatomic pathology laboratories, offering practical evidence to inform leadership-oriented interventions.

## 1. Introduction

Burnout is a major psychosocial occupational hazard that burdens both individuals and organizations. The World Health Organization (ICD-11) defines burnout as the result of chronic workplace stress that has not been successfully managed, characterized by emotional exhaustion, mental distancing from one’s work, and reduced professional efficacy [1]. Burnout is particularly relevant among healthcare professionals, who often work under sustained high demand. Ιn hospitals, work is inherently demanding, both physically and psychologically.

Professionals operate in high-intensity settings with constant time pressure, complex conditions, rising expectations from patients and management, excessive workloads, staffing shortages, emotional stress, and demanding schedules [2,3]. While caring for others can be fulfilling, over time it may lead to emotional exhaustion, cynicism, and a sense of professional ineffectiveness [4]. The consequences of burnout extend beyond the individual. Burned-out professionals tend to become disengaged from their work, deliver lower-quality services, report lower patient satisfaction, and have higher rates of medical errors. For these reasons, organizational leaders should prioritize physician well-being and recognize it as a quality indicator that is often overlooked [4,5]. The U.S. Agency for Healthcare Research and Quality estimates that burnout affects 10–70% of nurses and 30–50% of physicians [6], and the COVID-19 pandemic exacerbated the problem [3].

### 1.1. Burnout in Pathology Laboratories

Laboratory medicine, especially anatomic pathology, has been less explored regarding occupational burnout, despite its central role in diagnosis and its many prognostic and therapeutic indicators. Recently, rising case volume and complexity due to population ageing, emerging diseases, and an expanding diagnostic arsenal have further burdened teams that are often under intense pressure [7]. Staff are also exposed to chemical and biological hazards, while chronic understaffing of specialized roles and limited support for professional development hinder smooth operation and their well-being [8,9]. Beyond workload, organizational factors including demanding protocols, administrative/bureaucratic tasks, information systems poorly aligned with pathology workflows, work isolation, and constrained resources and infrastructure add to the strain [7,10,11,12]. Expectations for productivity and pressure to deliver a “definitive diagnosis” further exacerbate burnout, combined with limited managerial understanding/support and lower appreciation and recognition relative to other professionals with similar training [10,11,12,13]. For trainees and fellows, extra stressors include fear of diagnostic error, the need to appease senior staff, perceived inadequate preparation, and insufficient structured supervision/mentoring. These are factors that increase burnout risk, especially in academic centers with a complex case mix and educational/research obligations [7,13]. As organizational factors, these pathology-related conditions may contribute to emotional exhaustion, cynicism/depersonalization, and reduced personal achievement [7,14]. Recent evidence indicates a high prevalence of burnout among pathologists [15], although the self-reported nature of many studies may lead to underestimation so that actual burnout levels may be higher than published estimates [3]. Large surveys of laboratory personnel also link burnout to a strong intention to move or leave, with professionals primarily seeking better pay and benefits, improved location or conditions, work–life balance, and healthier work environments [10].

### 1.2. The Role of Leadership in Creating a Supportive Work Environment

In anatomic pathology laboratories, where demands for accuracy, tight deadlines, and organizational obstacles are high, leadership acts as a critical lever balancing demands and resources. Supervisor support and leadership are increasingly recognized as protective forms of organizational support that may mitigate burnout, reinforcing the relevance of examining leadership style in this work environment [14]. Selecting a leader trained in engagement and leadership, with an understanding of both laboratory practice and management, is key to a culture of well-being and fair resource allocation [7]. However, there is a gap in leadership training, even though increasing complexity requires physicians to play an active role in interdisciplinary decisions and policies [16]. Contemporary evidence links authentic, transformational, and servant leadership with lower burnout and stronger psychological resources, supporting leadership development as a preventive strategy [14].

In this work environment, leadership can influence burnout by shaping organizational conditions such as role clarity, autonomy, and supervisory support, which are central to everyday work practices. Among positive leadership styles, transformational leadership is often highlighted for its potential to mitigate burnout in healthcare professionals by encouraging engagement and mobilizing teams around a common purpose [17]. Authentic leadership has also been associated with better well-being, possibly through strengthening constructive psychological processes such as identification, hope, optimism, and trust [18]. In contrast, more controlling and authoritarian approaches appear to increase burnout, with evidence suggesting that their impact may manifest through a deterioration in organizational climate and a reduction in psychological capital [19]. Findings from laboratory settings recommend emphasizing transformational or situational leadership behaviors while minimizing passive/avoidant patterns, which may strengthen affective commitment to change and sustain a culture of continuous improvement [20].

For skills development, dedicated training programs provide managers with practical tools for staff support and a positive work environment [10]. In practice, leadership-scope interventions in laboratory settings have been associated with lower burden and inform local burnout-mitigation strategies [15,21]. Targeted investment in digital pathology and artificial-intelligence tools can reduce workload, support flexible/remote working, and improve efficiency [22]. Overall, supportive, values-aligned leadership through realistic, financially sustainable interventions that do not conflict with organizational goals can reduce burnout and improve the quality of services provided [23].

Despite this growing evidence, burnout in anatomic pathology remains underexplored compared with other healthcare domains. In Greece, and particularly in Attica, systematic data are limited, and the potential contribution of organizational factors such as leadership has not been sufficiently examined in this work environment. Accordingly, we conceptually treat perceived leadership as a proximal organizational factor expected to relate to burnout outcomes, consistent with evidence linking active/supportive leadership with lower burnout in healthcare settings.

## 2. Materials and Methods

This study aimed to (a) measure occupational burnout among staff in public, private, and university anatomic pathology laboratories in Attica, Greece; (b) determine the profile of leadership styles reported for laboratory heads; and (c) examine the relationship between leadership and burnout. The population was selected because, to our knowledge, no prior study in Greece or the European Union has combined the CBI and MLQ-5X specifically in anatomic pathology laboratories.

### 2.1. Sample

We conducted a cross-sectional study from December 2023 to April 2024 in anatomic pathology laboratories in Attica. This geographic focus facilitated access and helped keep approval procedures broadly consistent, while allowing the inclusion of public, private, and university-based settings within a single region. Attica also functions as the largest health-service hub in Greece, with pathology services that often support referral and second-opinion work beyond the metropolitan area, enhancing the policy and organizational relevance of this regional focus.

This study included all personnel at three public, two private, and two university anatomic pathology laboratories in Attica. Permission was obtained from the relevant institutional authorities at all participating sites. The study was approved by the Academic Program Committee of the Postgraduate Program in Health Care Management (DMY) at the Hellenic Open University, on 1 December 2023. Participation was voluntary, and all information provided was strictly confidential and anonymous, in accordance with the EU General Data Protection Regulation (GDPR) 2016/679. Written informed consent was obtained from all participants prior to completing the questionnaire.

Questionnaires were distributed to all eligible staff members, excluding the heads of the participating laboratories. The questionnaires were self-administered and provided in printed form. They were accompanied by an information letter outlining the study purpose and procedures. Completed questionnaires were returned to the research team in a single sealed envelope per laboratory. This procedure was chosen to protect anonymity, reduce social desirability bias, and encourage honest responses by preventing linkage of questionnaires to individual participants and reducing the risk of within-laboratory identification. Of 110 questionnaires distributed, 107 were returned (response rate 97.3%). None of the participating hospitals had a direct professional affiliation with any member of the research team to avoid any potential conflict of interest.

### 2.2. Research Instruments

Three questionnaires were used (a) to collect demographic data, (b) to assess the prevalence of occupational burnout, and (c) to determine the leadership model followed by each laboratory director.

(a) Demographic data: The first questionnaire, created by the research team, recorded participants’ demographic details: gender, age group, marital status, education level, employment setting (public, private, or university lab), type of employment, and years of experience in a pathology lab. Participants also indicated their professional role (physician, biologist, medical laboratory technologist, medical laboratory assistant, or administrative/secretarial staff). Finally, physicians were further classified by rank: those employed in the National Health System (NHS/ESY) included residents, auxiliary physicians, Registrar B, Registrar A, and directors (ESY rank); those in university labs included academic fellows, scientific associates, assistant professors, associate professors, and professors.
(b) Copenhagen Burnout Inventory (CBI): The second questionnaire we used was the CBI to measure the prevalence of occupational burnout. The instrument was developed by researchers at the National Institute of Occupational Health in Copenhagen [24] and originated from the Danish PUMA study (Project on Burnout, Motivation and Job Satisfaction), a 5-year longitudinal intervention launched in 1997 to examine burnout prevalence/distribution, causes, consequences, and interventions [24,25]

The CBI comprises 19 items across three sub-dimensions: personal burnout (PB, items PB.1–PB.6), work-related burnout (WB, items WB.1–WB.7), and client-related burnout (CB, items CB.1–CB.6). It has been widely used in many studies examining occupational burnout among healthcare professionals. [26,27,28,29,30,31,32].

In this study, we retained the original three-factor structure of the CBI but modified the third subscale to shift the focus from client-/patient-related burnout to colleague-related burnout. This adaptation consisted solely of replacing “clients/patients” with “colleagues” in the relevant items, with no other wording changes, while keeping the scale and scoring unchanged. Our approach aligns with prior applications across various domains [33,34,35,36,37,38,39,40]. Within anatomic pathology laboratories, a similar approach has been used with another burnout assessment instrument [15].

Scoring at the item level is mapped to a 0–100 scale, following the scheme shown in Table 1 (item WB.7 is reverse-scored). At the scale level (PB, WB, CB), scores are calculated as the mean of the relevant item scores for each burnout dimension. In addition, the overall burnout score (OB) is given by the mean across all items.

The CBI is freely available from its developers [24]. The Greek translation and psychometric validation in a Greek sample were conducted by Papaefstathiou et al. (2019) [41], from whom permission was obtained to use the version employed in the present study.

(c) Multifactor Leadership Questionnaire (MLQ-5X): The third questionnaire used to determine the leadership model followed by each laboratory director is the third edition of the Multifactor Leadership Questionnaire (MLQ-5X). The instrument was originally developed in 1995 and has undergone two revisions, with the latest (2004) remaining in use to date [42,43]. The MLQ-5X has been widely used in research both in healthcare organizations [20,44,45,46,47,48] and in other sectors [49,50]. The MLQ includes 45 descriptive items. Participants rate how often each statement describes their department head using the following response categories: 0 = “Not at all”, 1 = “Once in a while”, 2 = “Sometimes”, 3 = “Fairly often”, 4 = “Frequently, if not always” (numeric values indicate scoring). The statements form nine subscales that correspond to three leadership style profiles: Transformational, Transactional, and Passive/Avoidant. Additionally, the MLQ includes three outcome variables: Extra Effort, Effectiveness, and Satisfaction, which reflect consequences of the leadership style (Table 2). Subscale and outcome scores were computed as the mean of the corresponding item ratings (0–4), following the official scoring instructions, with higher scores indicating greater levels of the construct. The licence to use the MLQ-5X was obtained from Mind Garden, Inc (Menlo Park, CA 94026 USA).

### 2.3. Statistical Analysis

Before analysis, the dataset was screened for missing values and out-of-range responses; no missing data were detected. Distributions of questionnaire responses are presented as relative frequencies at the item level for the CBI and aggregated within each MLQ-5X subscale and outcome. Response category frequencies have been visualized using heat maps complemented by item micro-charts (mini histograms). Internal consistency reliability was assessed using Cronbach’s alpha at both the scale and subscale levels and interpreted using commonly applied research thresholds (poor/questionable/acceptable/good/excellent), with indicative cut-offs at 0.50, 0.60, 0.70, 0.80, and 0.90. Additionally, the ordinal alpha based on polychoric correlations was computed to provide a complementary reliability estimate for ordinal (Likert-type) items. Specifically, for the colleague-related burnout subscale (adapted from the original client-related burnout domain), internal consistency evaluation was supplemented with “alpha if item deleted”, inter-item correlations, and corrected item total correlations.

Scale and subscale scores were calculated according to each instrument’s scoring norms and summarized using the mean, standard deviation, median, minimum, maximum, and range. Mean values are presented with BCa 95% bootstrap confidence intervals (5000 resamples). Distributional assumptions were assessed using the Shapiro–Wilk test, skewness, and kurtosis indices and visual inspection of Q–Q plots; score distributions are additionally visualized using boxplots and normalized histograms overlaid with kernel density curves.

The primary exploration of potential linear associations was conducted using Pearson’s correlation coefficient (r). For each correlation, 95% BCa bootstrap confidence intervals (5000 resamples) were computed, with *p*-values reported for completeness. Differences across related outcomes were tested using the Friedman test (e.g., comparing the three CBI subscales and the MLQ-5X style composites within participants). When significant, pairwise comparisons were performed using Wilcoxon signed-rank tests with Bonferroni adjustment; Kendall’s W was reported as an effect size. For comparisons across independent tenure groups (five categories), one-way ANOVA with polynomial contrasts (linear and quadratic) was used when assumptions were acceptable. The overall main effect (omnibus test) of tenure was evaluated first, and polynomial contrasts were then used to assess linear and quadratic trends across the ordered tenure categories. Τhe Kruskal–Wallis test was used as a non-parametric check, with Bonferroni-adjusted pairwise Mann–Whitney tests when applicable.

Associations between leadership styles and burnout outcomes were examined using hierarchical linear multiple regression with forced-entry blockwise variable inclusion. Demographic variables (gender, specialty, and employment type) were entered simultaneously in Block 1, followed by perceived leadership style scores in Block 2. The incremental contribution of leadership styles was evaluated using changes in explained variance (ΔR^2^) and corresponding F-tests. Regression results are reported using unstandardized (B) and standardized (β) coefficients; in addition to conventional inference, coefficient estimates and *p*-values were complemented with BCa bootstrap calculations (5000 resamples). Multicollinearity was assessed via tolerance and variance inflation factor (VIF) statistics (tolerance > 0.20; VIF < 5), and independence of errors was evaluated using the Durbin–Watson statistic (approximately 1.5–2.5); all diagnostics were within acceptable limits. Linearity and homoscedasticity were assessed using residuals-versus-fitted plots, and influential observations were screened using Cook’s distance. Because no missing data were present, all models were estimated on the full sample (*N =* 107).

All tests were two-tailed with α = 0.05. Analyses were conducted in [SPSS 29.0.1.0] and R ([4.3.3]; packages: psych, lavaan, boot).

## 3. Results

### 3.1. Sample’s Characteristics

Participant characteristics are summarized in Table 3; the sample was predominantly female, just over half were permanently employed, and about one third had up to 5 years of work experience.

### 3.2. Internal Consistency

Table 4 summarizes internal consistency for all study scales and subscales using Cronbach’s alpha (α). For the CBI, overall burnout (OB) and the domain-specific subscales (PB, WB, CB) showed very good to excellent reliability (α = 0.88–0.92). For the MLQ-5X, reliability at the scale level was excellent for transformational leadership (α = 0.92) and very good for passive/avoidant leadership (α = 0.88), whereas transactional leadership was borderline acceptable (α = 0.70). At the subscale level, the two four-item transactional facets (CR, MBEA) yielded lower alpha values (approximately 0.60), and the IC facet also showed low internal consistency (α = 0.57). Item-level diagnostics indicated that only marginal improvements were achievable for the transactional scale and its facets. In contrast, IC could be improved (α = 0.72), with a slight increase in overall transformational leadership (α = 0.93), if one specific item were removed; however, all items were retained to preserve comparability with standard MLQ-5X scoring, given that transformational leadership scores were computed as the mean of 20 items and the influence of any single item on the composite score would therefore be limited. Furthermore, the higher α for transformational leadership should be considered in the context of its larger number of items (n = 20 items), which tends to yield higher internal consistency estimates. The ordinal alpha based on polychoric correlations was also computed, as it is often recommended for reliability estimation with ordinal response scales.; coefficients were consistently higher than Cronbach’s alpha but did not alter the overall pattern across scales and subscales.

### 3.3. Colleague-Related Burnout (Adaptation)

The internal consistency of the adapted colleague-related burnout subscale (derived from the original client-related burnout domain) was evaluated not only at the scale level (Cronbach’s α) but also using item- and scale-level diagnostics, as shown in Table 5. The results show that Cronbach’s α “if item deleted”, inter-item correlations (pairwise and mean), and corrected item–total correlations were generally within commonly used ranges indicative of acceptable-to-good internal consistency.

### 3.4. Copenhagen Burnout Inventory (CBI)

Table 6 presents item-level response distributions across the five rating categories (0/25/50/75/100) for all 19 CBI items (columns are aligned by assigned rating across items, with the reverse-scored WB.7 displayed accordingly). To highlight items with greater response dispersion, we identified, within each subscale, the items with the highest combined endorsement of the two lowest (0/25) and two highest (75/100) categories. These were PB.1 (“How often do you feel tired”; 60.7%) and PB.6 (“How often do you feel weak and susceptible to illness”; 51.5%) for personal burnout; WB.4 (“Do you feel worn out at the end of the working day”; 43.9%) and WB.3 (“Does your work frustrate you”; 58.0%) for work-related burnout; and CB.4 (“Do you feel you give more than you get back when working with your colleagues”; 34.6%) and CB.2 (“Do you find working with your colleagues frustrating”; 71.1%) for colleague-related burnout.

At the subscale level (pooled across items), category distributions were as follows: PB: 4.7%—22.8%—37.5%—29.9%—5.1%; WB: 11.5%—28.5%—30.3%—22.2%—7.6%; and CB: 33.5%—28.4%—22.4%—12.0%—3.7%. When categories were collapsed, the pooled top two (75/100) shares were 35.0% (PB), 29.8% (WB), and 15.7% (CB), whereas the pooled bottom two (0/25) shares were 27.5% (PB), 40.0% (WB), and 61.9% (CB). Overall, PB and WB showed higher concentrations in the upper categories than CB, whereas CB showed the highest concentration in the lower categories, with WB intermediate between the two. The heatmap visually reinforces this pattern, with darker shading in the personal and work-related domains and lighter shading for colleague-related interactions.

### 3.5. CBI Scales Scoring

Next, we computed participant-level scores on a 0–100 metric (higher values indicate greater burnout) by averaging the items within each subscale, and an overall CBI score by averaging all 19 items. Table 7 summarizes the main descriptive statistics for these scores. As shown, personal and work-related burnout were in the mid range, whereas colleague-related burnout was clearly lower. The Shapiro–Wilk tests, inspection of Q–Q plots, and distributional indices indicated that the personal, work-related, and overall burnout scores were approximately normally distributed, whereas colleague-related burnout showed a significant deviation from normality, with a modest right skew.

A related-samples Friedman test comparing the three subscales was statistically significant (*p* < 0.001), indicating differences in burnout profiles across domains. Bonferroni-adjusted Wilcoxon signed-rank tests showed that personal burnout was higher than both work-related burnout (adjusted *p* = 0.003) and colleague-related burnout (adjusted *p* < 0.001), and work-related burnout was higher than colleague-related burnout (adjusted *p* < 0.001), yielding the ordering PB > WB > CB.

**Figure 1:**
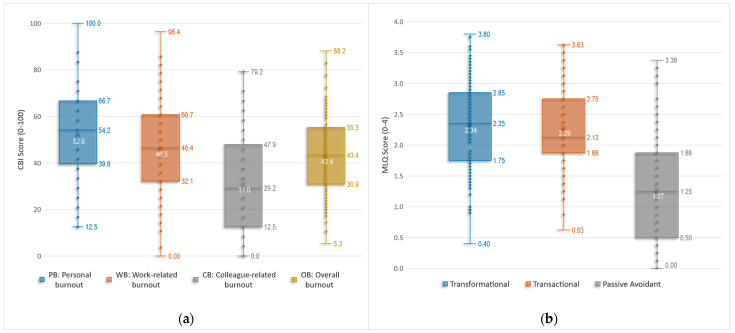
Distributions of burnout and leadership scores via box-and-whisker plots. (**a**) Copenhagen Burnout Inventory subscales—Personal (PB), Work-related (WB), Colleague-related (CB)—and Overall Burnout (OB), scored 0–100. (**b**) MLQ leadership styles—Transformational, Transactional, and Passive/Avoidant—scored 0–4. Numeric labels show the five-number summary (min, Q1, median, Q3, max); the ‘×’ marks the mean (its value is shown in white).

**Figure 2:**
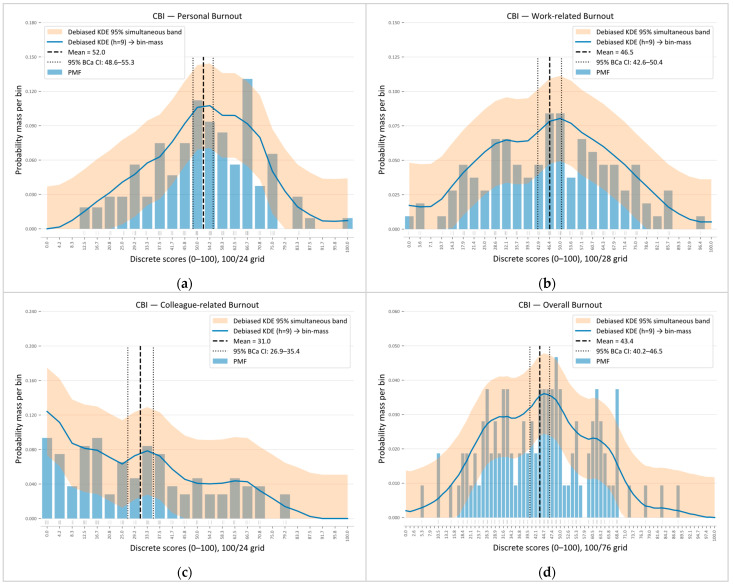
Distributions of Copenhagen Burnout Inventory scores. Panels show (**a**) Personal, (**b**) Work-related, (**c**) Colleague-related, and (**d**) Overall burnout (0–100 scales). Bars depict the empirical probability mass function (PMF) on the corresponding discrete grids (100/24, 100/28, 100/24, 100/76). The solid blue line is a debiased (bias-corrected) Epanechnikov KDE (bandwidth ℎ = 9) mapped to bin mass; the orange band is its simultaneous 95% confidence band. The vertical dashed line marks the sample mean, and the dotted lines the BCa 95% bootstrap CI for the mean.

The corresponding score distributions are shown in Figure 1a as boxplots and in Figure 2 as normalized histograms. The histogram bins were aligned with each scale’s discrete scoring grid (bin widths of 100/24 for PB, 100/28 for WB, 100/24 for CB, and 100/76 for overall burnout, OB). In both visualizations, personal and work-related burnout cluster around the middle of the scale, with approximately symmetric distributions and moderate spread, indicating variability among respondents. Colleague-related burnout is shifted towards lower values and shows a slight right skew, with most respondents reporting low-to-moderate CB and very high scores being uncommon. The overall burnout score lies in the mid–to-lower range (mean 43.4) with modest skew.

### 3.6. Multifactor Leadership Questionnaire (MLQ-5X)

Table 8 presents response category distributions for the 45 MLQ-5X items, aggregated within the nine subscales and the three outcomes. Overall, transformational and transactional leadership subscales show responses concentrated in the mid-to-upper categories (“sometimes” to “frequently, if not always”), whereas the passive/avoidant subscales are concentrated in the lower categories (“not at all” and “once in a while”). The three outcome scales were predominantly rated in the mid-to-high categories, with effectiveness and satisfaction showing particularly high endorsement (most responses falling within the top three categories), followed by extra effort. The heatmap shading and item micro-charts further illustrate these shifts in response concentration across leadership styles, subscales, and outcomes.

### 3.7. MLQ Leadership Styles Scores

Table 9 summarizes the mean scores and descriptive statistics based on participants’ responses on the 0–4 scale (with higher values indicating greater endorsement of each construct). At the scale level, the distributions of the three MLQ leadership styles are depicted in the corresponding boxplots in Figure 1b. The Shapiro–Wilk tests, Q–Q plot inspection, and distributional indices suggest that the transformational and transactional leadership composites (TRF, TRN) are approximately normally distributed, whereas the passive/avoidant composite (PAS), several subscales (IA, IM, MBEP, LF), and all three outcome scales (EE, EFF, SAT) show statistically significant deviations from normality, despite generally small-to-moderate skewness.

A Friedman test showed overall differences across the three leadership styles, *χ*^2^(2) = 43.79, *p* < 0.001. On average, transformational leadership had the highest mean rank (2.31), followed closely by transactional leadership (2.21), whereas passive/avoidant leadership had the lowest rank (1.49). Bonferroni-adjusted post hoc tests indicated that passive/avoidant scores were significantly lower than both transformational and transactional leadership (both *p* < 0.001), while the two active styles did not differ significantly from each other (*p* = 0.452). Kendall’s W = 0.205 (*p* < 0.001) indicated a small-to-moderate degree of agreement in how participants ranked the three leadership styles, with passive/avoidant leadership generally rated lowest and the two active styles rated higher.

**Figure 3:**
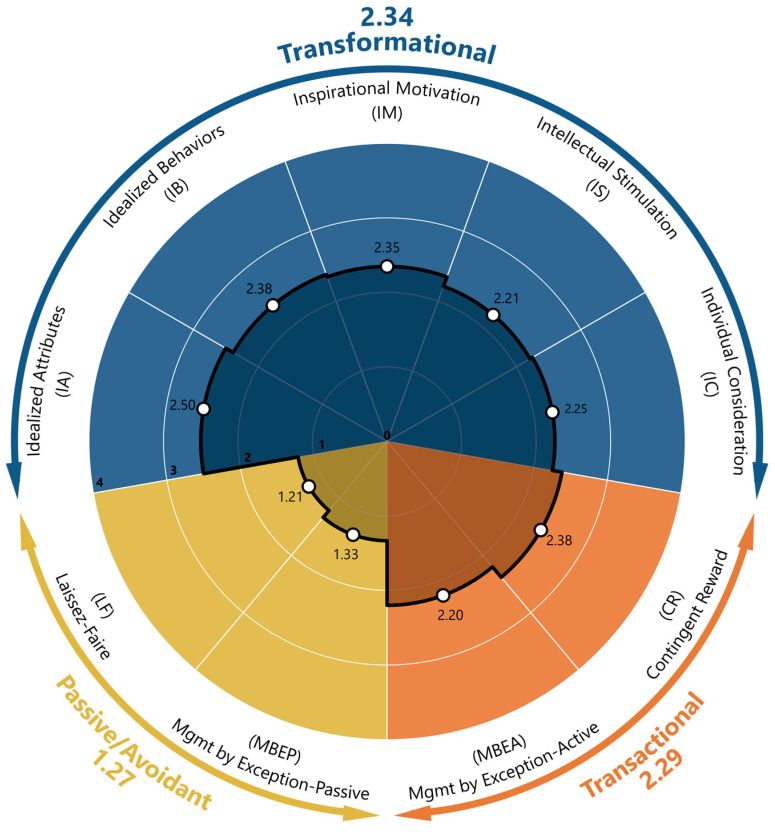
Radar plot of MLQ-5X leadership scores: Transformational (IA, IB, IM, IS, IC); Transactional (CR, MBEA); Passive/Avoidant (MBEP, LF). Colored sectors group subscales by style; concentric rings mark the radial 0–4 scale (origin at the center). White dots trace subscale means. Numbers on the outer arc report style-level means.

Furthermore, a related-samples Friedman test across the nine constituent subscales indicated differences in central tendency, *χ*^2^(8) = 121.60, *p* < 0.001, with small-to-moderate concordance (Kendall’s W = 0.142). The mean-rank profile suggested higher rankings for the most active/transformational facets—Idealized Influence–Attributed (IA; mean rank = 6.20), Contingent Reward (CR; 5.74), Idealized Influence–Behavior (IB; 5.70), and Inspirational Motivation (IM; 5.43)—with the remaining active facets showing similar mid-range ranks (Management by Exception–Active, Individualized Consideration, and Intellectual Stimulation; mean ranks ≈ 5.01–5.14). The lowest ranks were observed for the passive/avoidant facets—Management by Exception–Passive (MBEP; 3.50) and Laissez-faire (LF; 3.21). Post hoc Wilcoxon signed-rank tests with Bonferroni adjustment showed a consistent pattern; LF and MBEP did not differ from each other (adjusted *p* = 1.000) and were each significantly lower than all active facets (all adjusted *p* ≤ 0.002), whereas pairwise differences among the active facets (IA, IB, IM, IS, IC, CR, MBEA) were not significant after multiplicity control (all adjusted *p* ≥ 0.055). Overall, active (transformational/contingent reward) facets tended to receive higher mean ranks than passive/avoidant facets in this sample. Figure 3 (radar plot) depicts the overall structure of the derived leadership styles and their constituent subscales.

### 3.8. Burnout–Leadership Correlations

Using Pearson’s r with BCa 95% bootstrap confidence intervals (5000 resamples), we examined linear associations between burnout and leadership constructs and observed a broadly coherent pattern across burnout domains: As shown in Table 10, transformational leadership (TRF) consistently showed negative associations with all burnout dimensions (personal, work-related, colleague-related, and overall), indicating that higher transformational leadership scores were linked to lower burnout levels. This pattern was broadly mirrored at the subscale level: Idealized Attributes (IA) showed the strongest and most consistent inverse associations, while Intellectual Stimulation (IS) and Inspirational Motivation (IM) demonstrated similarly consistent negative relationships across burnout domains. Individualized Consideration (IC) also showed an inverse pattern but was weaker and less robust, particularly for personal burnout. Idealized Behaviors (IB) showed a similarly inverse but less robust pattern: only colleague-related burnout was supported (although the confidence interval included values close to zero), whereas personal and work-related burnout were not supported; accordingly, the association with overall burnout was only marginally supported.

Transactional leadership (TRN) followed the same inverse pattern as transformational leadership; however, the associations were weaker and less robust. Specifically, the relationship with personal burnout was not statistically significant, whereas the association with work-related burnout was only marginally supported, with clearer inverse associations observed for colleague-related and overall burnout. At the subscale level, Contingent Reward (CR) and Management by Exception—Active (MBEA) exhibited a comparable pattern.

A markedly different pattern emerged for passive/avoidant leadership (PAS), which was positively related to burnout across all dimensions. Both passive/avoidant subscales (Management by Exception—Passive and Laissez-Faire) were consistently associated with higher burnout. Among the burnout domains, the strongest associations were observed for colleague-related burnout, whereas the weakest were observed for personal burnout; similarly, overall burnout showed a consistently positive association.

Regarding MLQ outcomes, Effectiveness and Satisfaction were consistently inversely associated with burnout across domains. In contrast, Extra Effort showed largely non-significant associations, apart from a marginal association with colleague-related burnout.

Overall, the results show a consistent pattern in which transformational leadership and favorable leadership outcomes are associated with lower burnout, whereas passive/avoidant leadership is associated with higher burnout. Transactional leadership exhibits comparatively weaker and less robust associations. Among burnout dimensions, colleague-related burnout shows the most robust associations with all MLQ constructs (leadership styles, subscales, and outcomes).

### 3.9. Correlations with Demographic and Other Characteristics

Correlations between burnout and participant characteristics were generally modest (Table 11). Gender was positively associated with personal and work-related burnout, whereas the association with colleague-related burnout was not supported. The association between gender and overall burnout was weak and not robust. Employment type was inversely associated with personal burnout, indicating lower personal burnout among non-permanent staff than among permanent staff. Age, marital status, specialty, educational degree, and tenure showed no clear linear associations with any burnout domain.

Likewise, leadership styles and outcomes were largely unrelated to demographic characteristics (Table 12). Marital status showed modest inverse associations with transformational and transactional leadership, whereas tenure was inversely associated with extra effort. All remaining correlations were small and not statistically robust.

Furthermore, given the ordinal nature of the tenure variable, we first tested tenure-group differences in the three MLQ style composites using Kruskal–Wallis tests, which indicated differences for transformational (*p* = 0.024) and transactional leadership (*p* = 0.032) but not passive/avoidant leadership (*p* = 0.178). We then used one-way ANOVA with polynomial contrasts to characterize the form of these effects. Tenure was significantly associated with transformational leadership, F(4, 102) = 3.006, *p* = 0.022, and transactional leadership, F(4, 102) = 2.990, *p* = 0.022, with both patterns driven by significant negative quadratic components (TRF *p* = 0.005; TRN *p* = 0.006), consistent with concave (inverted-U–type) profiles. For transformational leadership, however, the linear component was also significant (*p* = 0.046), indicating an overall downward trend superimposed on the concave curvature—i.e., an inverted U-profile with a negative tilt. In both leadership profiles, peaking (local maximum) occurred within the intermediate tenure categories rather than at the extremes. Passive/avoidant leadership showed no significant omnibus effect, F(4, 102) = 1.348, *p* = 0.257, and only a borderline quadratic component (*p* = 0.050), indicating weak and non-robust curvature.

As for the three outcomes, tenure in the current position showed distinct patterns. For extra effort (EE), a one-way ANOVA indicated a significant tenure effect, F(4, 102) = 3.77, *p* = 0.007, driven by a strong negative linear contrast, t(102) = −3.51, *p* < 0.001, with a small non-significant quadratic component (*p* = 0.066); this monotonic decline is consistent with the previously observed negative Pearson correlation between tenure and EE (Table 8). For effectiveness (EFF), the ANOVA was also significant, F(4102) = 3.35, *p* = 0.013, with a robust negative quadratic contrast, t(102) = −3.33, *p* = 0.001, but no clear linear trend (*p* = 0.089), suggesting an inverse U-shaped pattern with peak effectiveness at intermediate tenure levels. In contrast, satisfaction with the leader (SAT) showed no meaningful tenure gradient, with a non-significant ANOVA, F(4, 102) = 0.81, *p* = 0.520, and non-significant polynomial contrasts, indicating that satisfaction ratings were broadly similar across tenure groups.

### 3.10. Multiple Linear Regression

To examine the unique contribution of demographics and perceived leadership styles to burnout, we conducted hierarchical multiple linear regression analyses. In the first (baseline) block, gender, specialty, and employment type were entered simultaneously; in the second block (PB/WB/CB/OB-2), the three MLQ leadership style composites (transformational, transactional, passive/avoidant) were added. Separate models were estimated for personal, work-related, colleague-related, and overall burnout; results are presented in Table 13 (PB-1/2, WB-1/2) and Table 14 (CB-1/2, OB-1/2).

For personal burnout (PB), demographics accounted for a modest proportion of variance, with gender and employment type showing significant associations. The addition of leadership styles produced only a small, non-significant increment in explained variance (ΔR^2^), and none of the leadership style composites showed a unique association with PB in the mutually adjusted model. For work-related burnout (WB), the demographic block provided only weak evidence of association; however, model fit improved significantly after adding leadership styles, indicating that leadership styles as a set contributed beyond demographics. Nevertheless, no single leadership style composite retained a unique effect when entered simultaneously.

A different pattern was observed for colleague-related burnout (CB) and overall burnout (OB). After adjustment for demographics, passive/avoidant leadership showed a clear, unique positive association with both CB and OB, whereas transformational and transactional leadership did not retain unique effects in the mutually adjusted models. In the CB model, specialty also became an independent predictor after adjustment, with non-physician staff reporting higher colleague-related burnout than physicians.

Overall, demographics accounted for most of the explained variance in PB, leadership styles improved the WB model at the block level, and passive/avoidant leadership showed the most consistent unique associations with CB and OB. Across models, effect sizes were modest, and diagnostic checks were acceptable (Durbin–Watson = 1.85–2.01; no evidence of problematic multicollinearity based on tolerance and VIF statistics).

## 4. Discussion

Pathology laboratories operate under conditions of precision, strict documentation, and tight deadlines, where even minor errors can have outsized clinical effects. Over the past decade, the increase in the volume and complexity of cases combined with demanding procedures and protocols and tight turnaround times has created a high-pressure environment [7,10,11,22]. Furthermore, as professionals without direct patient contact, pathology laboratory workers are likely to have distinct risk factors and different priorities for intervention for burnout compared to direct-contact clinical teams [12]. However, despite the crucial role of laboratories in the diagnostic pathway, studies that systematically assess burnout among laboratory staff—and in particular, its relationship with leadership —remain limited. To our knowledge, the current study is among few in Greece and Europe to examine burnout in anatomic pathology laboratories across public, private, and university settings within a single regional hub. In this context, this study characterizes burnout levels among staff in anatomic pathology laboratories in Attica, Greece, and explores its association with leadership styles based on the MLQ-5X, focusing on actionable dimensions that can be targeted for intervention.

In our sample, overall burnout levels were moderate to low, with personal burnout being the highest, work-related burnout intermediate, and colleague-related burnout the lowest. The iternational literature on clinical laboratory staff reports a substantial burnout burden, with estimates varying significantly by context and methodology [7,51]. Canadian data show a high prevalence of burnout among pathologists [15], while national and local surveys from other countries also report high levels of burnout and overload-related fatigue among laboratory and pathology professionals [51,52].

To contextualize the magnitude of burnout in our sample, we compared our CBI scores with available international healthcare data. Compared with the international literature using the CBI, our average scores (personal 52.0, work-related 46.5) fall within the middle range. In Europe, personal burnout has been reported from 45.3 (Lithuania) to 58.9 (Latvia), with Greece at 50.1 and Ireland at 53.6; for work-related burnout, from 46.4 (Lithuania) to 56.0 (Latvia), with Greece at 52.9 and Ireland at 52.7 [26,28,31,32]. Outside Europe, averages in a similar range have also been reported, including in Lebanon, Arkansas, and Saudi Arabia [27,29,30]. Taking into account variation by country, occupational group, and timing (e.g., post-COVID-19), our profile suggests moderate-to-high personal burnout and moderate work-related burnout at levels comparable to many European and international cohorts.

Burnout related to colleague interactions was significantly lower than personal burnout, which may reflect the particularities of anatomic pathology as a non-patient-facing discipline [12]. At the same time, the central role of pathologists in multidisciplinary teams makes effective two-way communication with clinicians critical, yet this is often hampered by limited direct interaction and differing terminology [16,53]. Clinicians may find it difficult to assess the quality of a pathologist’s work and value direct telephone availability for ad hoc discussions of difficult cases or sampling/reporting issues [54]. Despite the lower colleague-related load, disruptive behaviors have been linked to increased work stress across laboratory staff, while perceptions of reduced professional recognition remain common [10,12,13,55]. Although colleague-related burnout was lower, it may have been influenced by the quality of interdisciplinary communication and work relationships.

It is worth noting that in our sample, more than half of the participants scored 50 or higher on the 0–100 burnout scale for the item on having enough energy for family and friends, indicating at least a moderate level of reduced leisure-time energy.

This finding is consistent with studies in laboratory settings, where work–life balance consistently emerges as a central challenge associated with an increased likelihood of burnout [10,13,51] and is an important correlate of work performance [3]. Among the factors that lead to burnout and reduce work–life balance, workload has been identified as a key driver [13], as it often increases quietly without a corresponding increase in human resources [22], with extra work potentially being absorbed into personal time [7]. The situation may be exacerbated by the absence of a standardized, internationally accepted workload index for fair allocation and evidence-based staffing planning, resulting in excessive workload that is not adequately captured by standard measures [7,56]. At the same time, limited use of formal administrative support as a source of social support has been reported [13], while those reporting better work–life balance tend to adopt practices such as not taking work home, regular exercise, hobbies, and structured personal time [10].

Burnout in pathology laboratories is not just a matter of well-being; it has been linked to diagnostic accuracy and patient safety [13]. Cognitive fatigue in high-workload environments where workload escalates with each incoming sample and there is no natural upper limit has been associated with increased risk of quality degradation and diagnostic errors, as well as reduced productivity, absenteeism, and additional operating costs [7,57]. At the individual level, emotional exhaustion may erode attention to detail and may impair decision-making in sensitive, complex cases [11].

Regarding gender, women in our sample reported higher personal and work-related burnout in bivariate analyses, with no meaningful gender differences in colleague-related or overall burnout. In multivariable regression analysis, after covariate adjustment, female gender remained an independent predictor only for personal burnout, suggesting that gender differences are most pronounced at the personal level and are attenuated after adjustment. This trend is consistent with findings from pathology departments showing greater emotional distress in women [11,15] and with reports of higher perceived stress among women in laboratory medicine [58]. Possible mediating factors include difficulties with work–life balance and the “double role” [14], as well as lower perceived control over workload, time for documentation, or alignment of values with management [12]. Additionally, permanent employment status was associated with higher personal burnout; this finding should be interpreted with caution, as it may reflect roles with greater responsibility and requires further investigation.

In bivariate analyses, specialty (physician vs. other staff) was not significantly correlated with any burnout dimension. However, in the multivariable regression model that adjusted for gender, employment type, and leadership styles, specialty emerged as an independent predictor of colleague-related burnout, with non-physician staff reporting higher levels of colleague-related burnout than physicians. One plausible interpretation consistent with the literature is that despite intense pressure to deliver a “final diagnosis” in a largely manual, less-automated field [12] and burdens from administrative dysfunctions and constant multitasking [52], pathologists may maintain a more horizontal network of interactions (e.g., cross-departmental collaborations), with greater autonomy and control, factors that may mitigate peer friction. By contrast, other laboratory personnel, including technologists and assistants, often report lower pay, less prestige, reduced recognition, and higher exposure to negative workplace behaviors, and they show a greater likelihood of burnout than physicians [12], with very high prevalence in some settings [7,11].

Recent evidence suggests that medical laboratory professionals may experience particularly pronounced institution-imposed workload and reduced control/autonomy [12]. Although these studies address overall burnout, they may help contextualize the higher colleague-related burden observed among non-physician staff in our sample, which may be related to more hierarchical working relationships and less influence in day-to-day decision-making. At the same time, the absence of differences in other burnout dimensions between physicians and non-physician personnel suggests that both groups face similar structural demands.

Regarding the other demographic variables, no statistically significant differences in burnout were found. However, the international literature notes some trends. In the limited evidence from anatomic pathology laboratories, unmarried individuals may score higher than married ones, with the highest burden often reported in ages 35–44 and the lowest among older individuals [11,15]. In addition, senior and academic pathologists, as well as those working in larger laboratories, tend to report higher job satisfaction, possibly due to better infrastructure or the achievement of professional goals [52]. Similarly, Canadian data on medical laboratory technologists (MLTs) show that older age and higher educational attainment are associated with a lower likelihood of burnout [51]. Collectively, these heterogeneities suggest that age, career stage, marital status, education, and institutional context can shape risk profiles, although these patterns were not supported with statistical significance in the present sample.

In our sample, active leadership styles (transformational/transactional) were rated significantly higher than passive/avoidant, while no statistically significant difference was observed between transformational and transactional leadership. This pattern is consistent with the view that transformational leadership builds on transactional leadership; it interacts with and reinforces it to achieve leaders’ and organizations’ goals [45]. Comparative studies in clinical settings likewise report a predominance of active styles: primarily transformational, followed by transactional, with passive/avoidant behaviors lowest [44]. Other work notes that transactional leadership is more common in day-to-day practice, with transformational leadership applied less often [47].

Self-assessment studies indicate the use of both transformational and transactional behaviors, while laissez-faire is rare, supporting the coexistence of these two leadership styles in practice [59]. Relatedly, older/more experienced supervisors tend to show stronger transformational traits, whereas younger supervisors score higher on passive/avoidant traits [60]. Recent studies also suggest leaders view transformational approaches as more effective than purely transactional or avoidant ones [61,62]. In summary, our findings accord with the international trend. Active styles (transformational/transactional) dominate and coexist, while passive/avoidant styles are less frequent. However, subordinate’s ratings are shaped by implicit leadership theories favoring behaviors aligned with their preferences and by evaluator characteristics (e.g., experience, accountability context, personality, mood), which warrants caution in interpretation [48,63].

Furthermore, regarding sample characteristics, no differences were found in leadership scores (MLQ–transformational, transactional, passive/avoidant) by gender, employment type (long-term/permanent versus other contracts), or profession (physicians versus other staff). Marital status was associated with more active styles higher transformational/transactional and lower passive/avoidant among married/civil-partnership individuals, without implying causality. However, tenure in the current position was associated with perceptions of active leadership, in a nonlinear manner. Both transformational and transactional leadership followed a significant inverse U-shaped pattern, with higher scores among staff with intermediate tenure and lower scores in the shortest- and longest-tenure categories. In contrast, passive/avoidant leadership did not exhibit robust tenure differences; the omnibus tests were nonsignificant, and the quadratic contrast was only borderline, so any apparent curvature in the profile plot should be interpreted with caution. A plausible interpretation is that as tenure increases, emphasis on transactional mechanisms (contingent reward) decreases [62], and the way staff perceive signals of inspiration and guidance changes; routine and role fatigue may weaken both transformational messages and transactional interactions. By contrast, mid-career staff more readily recognize a mix of vision/motivation and organization, action, performance measurement, and rewards.

Specifically, regarding staff perceptions of leadership across tenure groups, prior research shows that leaders’ intended practices often diverge from what staff experience, and that only perceived leadership is consistently linked to organizational performance [63]. Furthermore, behaviors aligned with team values and needs (e.g., differing priorities at mid- versus long-tenure stages) are more acceptable and effective. In contrast, misalignment reduces motivation and performance [64]. Finally, nursing data do not show consistent generational differences (Boomers vs. Gen X) in perceptions of managers’ leadership style; both groups more frequently recognized transformational and transactional behaviors than passive ones [65]. This suggests that tenure-related differences in our sample are more likely tied to role context and career stage than to chronological age and should be understood accordingly.

Tenure in current position also showed distinct associations with the three outcomes; extra effort declined with increasing tenure, effectiveness followed an inverse U-shaped pattern peaking at intermediate tenure, and satisfaction remained largely stable. Overall, tenure was more closely related to perceived effort and effectiveness than to satisfaction, suggesting a mid-career peak in effectiveness alongside a gradual reduction of extra effort over time. Within this broader organizational context, leadership style was also strongly associated with burnout outcomes.

In our study, professional burnout among anatomic pathology staff was positively associated with passive/avoidant leadership (PAS), including both of its facets, management by exception–passive and laissez-faire, and it was inversely associated with transformational leadership and more weakly with transactional leadership.

This aligns with evidence that transformational leadership correlates with higher satisfaction, perceived leader effectiveness, role/mission clarity, and open communication [66]. These patterns are consistent with previous studies in healthcare showing that laissez-faire and MBEP are associated with higher burnout among healthcare personnel [67] and that emotional exhaustion mediates the relationship between laissez-faire and burnout [68].

Recent studies in healthcare confirm the positive association between laissez-faire leadership and stress and intention to leave, whereas transformational leadership is negatively associated with these outcomes [69,70]. Similarly, findings indicate that transformational leadership is negatively related to burnout and positively to well-being/retention, providing protective effects in demanding environments [17,67,71]. Although evidence specific to anatomic pathology remains limited, the broader healthcare literature supports the relevance of leadership style in comparable clinical settings and underscores the gap the present study helps address.

In laboratory settings requiring continuous adaptation, transformational leadership is associated with a stronger culture of change and continuous improvement [20]. Compared with purely transactional approaches, transformational leadership places greater emphasis on individualized consideration, fostering closer relationships and greater organizational contribution [72]. Regarding job satisfaction, the link with leadership is well documented; transformational approaches correlate positively, whereas passive/avoidant and laissez-faire correlate negatively. Transactional leadership shows mixed associations depending on context [73].

Our study nevertheless has some limitations. First, it was based on a convenience sample from a small number of anatomic pathology laboratories in Attica, so the findings are not generalizable to all Greek settings. Moreover, convenience sampling implies non-random selection, which may introduce selection bias and limit external validity. Coverage within participating sites was near complete, which supports internal validity but does not resolve external validity concerns. Second, the cross-sectional, self-reported design (CBI, MLQ-5X) introduces risk of common-method bias and precludes causal inference. Accordingly, the observed associations should not be interpreted as evidence that leadership styles “cause” changes in burnout. Third, our adaptation of the CBI’s third subscale to assess “colleague-related” burnout limits strict comparability with published studies that are mainly based on the original client-facing wording. Additionally, leadership was measured only via employee perceptions and was not triangulated with leader self-ratings or objective indicators. Fourth, although the regression models included a limited number of predictors (six in total), statistical power for detecting smaller incremental effects may have varied by outcome (increasing the risk of Type II error), and model estimates may be sensitive to sampling variability in a modest sample size, so non-significant findings should be interpreted cautiously and confirmed in larger studies.

## 5. Conclusions

In this cross-sectional study of anatomic pathology laboratory staff in Attica, burnout levels were moderate to low overall, with personal burnout highest, work-related intermediate, and colleague-related lowest. A substantial proportion of respondents reported low energy for family or friends, suggesting a notable work–life strain in this sample. Differences between physicians and other laboratory personnel were mainly evident for colleague-related burnout in adjusted analyses. Active leadership styles were rated higher than passive/avoidant, with no significant difference between transformational and transactional leadership. Burnout was positively associated with passive/avoidant leadership dimensions, transformational leadership dimensions showed protective associations, whereas satisfaction with the leader was inversely associated with overall burnout. No significant differences in leadership scores were observed by gender, employment type, or profession. These findings highlight the potential value of strengthening active leadership behaviours and monitoring passive/avoidant patterns within anatomic pathology laboratories. Future studies with larger and more diverse samples should confirm these associations and explore causal pathways.

## Figures and Tables

**Table 1:** Copenhagen Burnout Inventory (CBI): subscales, item sets, and 0–100 scoring per response option.

CBI Burnout Scale	Items	Scoring Rate per Response				
		0	25	50	75	100
Personal burnout (PB)	PB.1-PB.6	Never	Seldom	Sometimes	Often	Always
Work-related burnout (WB)	WB.1-WB.3	To a very low degree	To a low degree	Somewhat	To a high degree	To a very high degree
	WB.4-WB.6	Never	Seldom	Sometimes	Often	Always
	WB.7	Always	Often	Sometimes	Seldom	Never
Colleague-related burnout (CB)	CB.1-CB.4	To a very low degree	To a low degree	Somewhat	To a high degree	To a very high degree
	CB.5-CB.6	Never	Seldom	Sometimes	Often	Always

**Table 2:** Structure of the MLQ-5X: leadership styles (scales), subscales, outcomes, abbreviations, and number of associated items.

Leadership Style (Abbreviation)	MLQ-5X Subscale/Outcome	Abbreviation	No. of Items
Transformational (TRF)	Idealized Attributes	IA	4
	Idealized Behaviors	IB	4
	Inspirational Motivation	IM	4
	Intellectual Stimulation	IS	4
	Individual Consideration	IC	4
Transactional (TRN)	Contingent Reward	CR	4
	Management by Exception–Active	MBEA	4
Passive/Avoidant (PAS)	Management by Exception–Passive	MBEP	4
	Laissez-Faire	LF	4
Outcomes	Extra Effort	EF	3
	Effectiveness	EFF	4
	Satisfaction with the Leader	SAT	2

Note: MLQ Form 5X-Short used under license from Mind Garden, Inc. Copyright © 1995 by Bernard Bass & Bruce J. Avolio. All rights reserved in all media. Published by Mind Garden, Inc.

**Table 3:** Participants’ characteristics (*N* = 107).

Characteristic	N	%	Characteristic	N	%
Gender			Employment type		
Male	39	36.4%	Permanent	59	55.1%
Female	68	63.6%	Non-permanent	48	44.9%
Marital status			Tenure (years)		
Married	60	56.1%	(0–5]	38	35.5%
Single	47	43.9%	(5–10]	18	16.8%
Specialty			(10–15]	18	16.8%
Doctors	47	43.9%	(15–20]	12	11.2%
Other Staff	60	56.1%	(20+]	21	19.6%

**Table 4:** Internal consistency of scales and subscales.

Scale (Subscale)	α	Scale (Subscale)	α	Scale (Subscale)	α	Scale (Subscale)	α
OB	0.92	TRF	0.92	TRN	0.70	PAS	0.88
(PB)	0.88	(IA)	0.87	(CR)	0.61	(MBEP)	0.69
(WB)	0.88	(IB)	0.67	(MBEA)	0.58	(LF)	0.85
(CB)	0.89	(IM)	0.85				
		(IS)	0.73				
		(IC)	0.57				

Note: α = Cronbach’s alpha; Scale/subscale abbreviations as per Table 2.

**Table 5:** CBI Colleague-related Burnout (adapted from the original client-related burnout subscale): item- and scale-level internal consistency diagnostics (*N* = 107).

CBI	Cronbach’s α	Inter-Item Correlations					Corrected Item-Total Correlations
Item	If Item Deleted	Mean	Min	(Min with)	Max	(Max with)	
CB.1	0.86	0.63	0.54	CB.4	0.73	CB.2	0.77
CB.2	0.87	0.60	0.44	CB.4	0.73	CB.1	0.71
CB.3	0.86	0.63	0.59	CB.2	0.69	CB.1	0.78
CB.4	0.90	0.51	0.44	CB.2	0.63	CB.3	0.60
CB.5	0.87	0.62	0.48	CB.4	0.77	CB.6	0.75
CB.6	0.87	0.60	0.44	CB.4	0.77	CB.5	0.72
Overall	0.89	0.60	0.44	CB.2–CB.4	0.77	CB.5–CB.6	0.72

Note: Item rows report α if the listed item is deleted, mean/min/max inter-item correlations of the row item with the remaining items (with corresponding items for min/max), and corrected item–total correlations. The “Overall” row reports scale-level α, mean/min/max inter-item correlations (with corresponding item pairs), and the mean corrected item–total correlation across items. All correlations are Pearson’s r.

**Table 6:** Copenhagen Burnout Inventory (CBI): item-level response distributions (percent) with heatmap shading, response wording, applicable scoring, and item micro-charts (IMC), (*N =* 107).

Questionnaire Item		Response Category (%)					IMC
	(f):	Never	Seldom	Sometimes	Often	Always	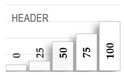
	(d):	To a Very Low Degree	To a Low Degree	Somewhat	To a Very Low Degree	To a Low Degree	
		(Rate = 0)	(Rate = 25)	(Rate = 50)	(Rate = 75)	(Rate = 100)	
PB.1 How often do you feel tired?	(f)	1.9	5.6	31.8	50.4	10.3	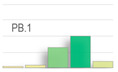
PB.2 How often are you physically exhausted?	(f)	2.8	15.0	41.1	36.4	4.7	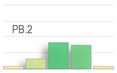
PB.3 How often are you emotionally exhausted?	(f)	0.9	25.2	40.2	26.2	7.5	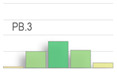
PB.4 How often do you think “I cannot take it anymore”?	(f)	10.3	31.8	32.7	21.5	3.7	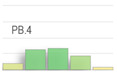
PB.5 How often do you feel worn out?	(f)	1.9	17.8	43.9	32.7	3.7	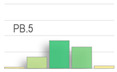
PB.6 How often do you feel weak and susceptible to illness?	(f)	10.3	41.2	35.5	12.1	0.9	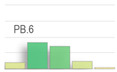
WB.1 Is your work emotionally exhausting?	(d)	10.3	17.8	33.6	28.0	10.3	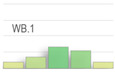
WB.2 Do you feel burnt out because of your work?	(d)	7.5	23.4	28.0	30.8	10.3	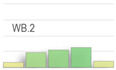
WB.3 Does your work frustrate you?	(d)	24.3	33.7	25.2	10.3	6.5	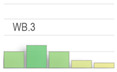
WB.4 Do you feel worn out at the end of the working day?	(f)	2.8	18.7	34.6	34.6	9.3	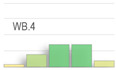
WB.5 Are you exhausted in the morning at the thought of another day at work?	(f)	13.1	36.5	27.1	16.8	6.5	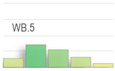
WB.6 Do you feel that every working hour is tiring for you?	(f)	9.3	36.5	27.1	20.6	6.5	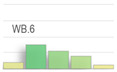
WB.7 Do you have enough energy for family and friends during leisure time?	(f*)	Always 13.1	Often 32.7	Sometimes 36.5	Seldom 14.0	Never 3.7	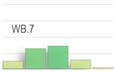
CB.1 Do you find it hard to work with your colleagues?	(d)	30.8	29.9	24.3	15.0	0.0	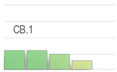
CB.2 Do you find working with your colleagues frustrating?	(d)	41.2	29.9	18.7	9.3	0.9	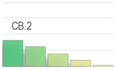
CB.3 Does working with your colleagues drain your energy?	(d)	33.7	28.0	26.2	9.3	2.8	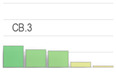
CB.4 Do you feel you give more than you get back when working with your colleagues?	(d)	23.3	23.4	18.7	16.8	17.8	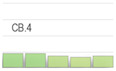
CB.5 Are you tired of working with your colleagues?	(f)	35.5	31.8	24.3	8.4	0.0	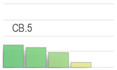
CB.6 Do you ever wonder how long you will be able to keep working with your colleagues?	(f)	36.5	27.1	22.4	13.1	0.9	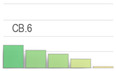

Note: For each item, cells show the percentage of respondents endorsing the corresponding category (row-wise). When needed, the largest category was adjusted by ±0.1 percentage points so that row totals equal 100%. Colors use a global 0–50.5% (maximum observed) scale (lighter = lower share, darker = higher). Item prefixes denote subscales: PB = Personal burnout; WB = Work-related burnout; CB = Colleague-related burnout. Superscripts (f)/(f*)/(d) mark the response wording: f = frequency (Never, Seldom, Sometimes, Often, Always → 0, 25, 50, 75, 100); f* = frequency (reverse order scoring)—applies to item WB.7; d = degree (To a very low degree, To a low degree, To some degree, To a high degree, To a very high degree → 0, 25, 50, 75, 100). Categories are consistently ordered left-to-right by assigned rating (0, 25, 50, 75, 100). Right-hand item micro-charts (IMC) display bar charts of the five-category shares for each item.

**Table 7:** Copenhagen Burnout Inventory (CBI) scores by subscale and overall, with summary statistics (*N =* 107).

CBI	Mean	BCa 95% CI		SD	Median	Min	Max	Range	Skewness/ Kurtosis	Shapiro–Wilk	
		LB	UB							W	*p*
Personal (PB)	52.0	48.6	55.3	17.7	54.2	12.5	100.0	87.5	−0.13/−0.25	0.99	0.29
Work-related (WB)	46.5	42.6	50.4	20.7	46.4	0.0	96.4	96.4	0.00/−0.55	0.99	0.59
Colleague-related (CB)	31.0	26.9	35.4	22.5	29.2	0.0	79.2	79.2	0.41/−0.87	0.94	<0.001
Overall (OB)	43.4	40.2	46.5	16.7	43.4	5.3	88.2	82.9	0.15/−0.36	0.99	0.71

Note: BCa 95% CI = bias-corrected and accelerated bootstrap confidence interval; LB/UB = lower/upper CI bounds. CIs are based on 5000 bootstrap resamples.

**Table 8:** Aggregated item response distributions (%) by MLQ-5X subscale and outcome with heatmap shading and item micro-charts (*N =* 107).

Leadership Style	Subscales and Outcomes	Response Category (%)					IMC
		Not at All	Once in a While	Sometimes	Fairly Often	Frequently, If Not Always	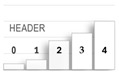
		(Rate = 0)	(Rate = 1)	(Rate = 2)	(Rate = 3)	(Rate = 4)	
Transformational (TRF)	IA	7.5	15.7	23.8	25.5	27.5	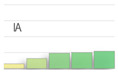
	IB	7.0	15.7	27.8	31.0	18.5	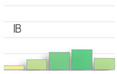
	IM	9.3	19.4	24.5	31.8	15.0	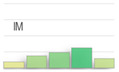
	IS	7.2	20.6	31.3	25.2	15.7	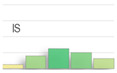
	IC	11.2	16.8	26.6	26.2	19.2	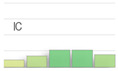
Transactional (TRN)	CR	8.4	17.5	22.9	29.7	21.5	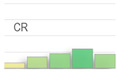
	MBEA	9.6	18.7	30.8	23.8	17.1	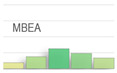
Passive/ Avoidant (PAS)	MBEP	31.9	26.9	22.2	13.6	5.4	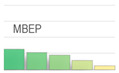
	LF	37.2	25.2	21.7	11.0	4.9	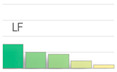
Outcomes	EF	13.1	13.4	31.7	23.1	18.7	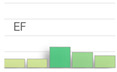
	EFF	4.9	8.6	28.7	28	29.8	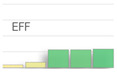
	SAT	4.2	9.3	25.2	30.9	30.4	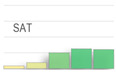

Note: Each cell shows the percentage of respondents endorsing the corresponding category (row-wise). When needed, the largest category was adjusted by ±0.1 percentage points so that row totals equal 100%. Colors follow a global scale across the observed min–max range (lighter = lower share; darker = higher share). IMC = item micro-chart.

**Table 9:** Descriptive statistics for MLQ-5X leadership styles, subscales, and outcomes (*N =* 107).

MLQ-5X Construct	Mean	SD	BCa 95% CI		Median	Min	Max	Range	Skewness/ Kurtosis	Shapiro–Wilk	
			LB	UB						W	*p*
Leadership Style											
TRF	2.34	0.75	2.20	2.48	2.35	0.40	3.80	3.40	−0.16/−0.58	0.99	0.33
TRN	2.29	0.67	2.16	2.42	2.13	0.63	3.63	3.00	0.03/−0.55	0.98	0.08
PAS	1.27	0.87	1.11	1.44	1.25	0.00	3.38	3.38	0.27/−0.82	0.96	<0.01
Subscale											
IA	2.50	1.05	2.30	2.70	2.75	0.25	4.00	3.75	−0.31/−0.83	0.95	<0.001
IB	2.38	0.80	2.23	2.53	2.50	0.25	4.00	3.75	−0.23/−0.50	0.98	0.08
IM	2.35	0.97	2.17	2.52	2.50	0.00	4.00	4.00	−0.32/−0.65	0.97	<0.01
IS	2.21	0.85	2.06	2.37	2.25	0.25	4.00	3.75	0.06/−0.26	0.98	0.20
IC	2.25	0.79	2.10	2.41	2.25	0.50	4.00	3.50	0.01/−0.57	0.98	0.21
CR	2.38	0.82	2.23	2.54	2.50	0.50	4.00	3.50	−0.01/−0.72	0.98	0.08
MBEA	2.20	0.77	2.05	2.35	2.25	0.00	3.75	3.75	−0.30/−0.24	0.98	0.09
MBEP	1.33	0.86	1.17	1.50	1.25	0.00	3.50	3.50	0.22/−0.74	0.96	<0.01
LF	1.21	0.98	1.04	1.39	1.00	0.00	4.00	4.00	0.43/−0.62	0.93	<0.001
Outcome											
EE	2.21	0.81	2.05	2.37	2.33	0.00	4.00	4.00	−0.46/−0.02	0.97	0.01
EFF	2.69	0.95	2.50	2.88	2.75	0.00	4.00	4.00	−0.32/−0.67	0.95	<0.001
SAT	2.74	0.90	2.57	2.91	3.00	0.50	4.00	3.50	−0.37/−0.54	0.94	<0.001

Note: Abbreviations for leadership styles and subscales are as in Table 2. Row values represent the mean of the listed subscales. Scores range 0–4 (higher = more of the construct). BCa 95% CI = bias-corrected and accelerated bootstrap confidence interval; LB/UB = lower/upper CI bounds. CIs are based on 5000 bootstrap resamples.

**Table 10:** Pearson correlations (*r*) between MLQ-5X leadership constructs and burnout (personal, work-related, colleague-related, overall) with 95% BCa bootstrap confidence intervals (*N* = 107).

MLQ-5X Construct	Burnout (CBI)											
	Personal			Work-Related			Colleague-Related			Overall		
	*r*	LB	UB	*r*	LB	UB	*r*	LB	UB	*r*	LB	UB
Leadership Style												
TRF	−0.24	−0.41	−0.05	−0.30	−0.47	−0.10	−0.34	−0.50	−0.17	−0.36	−0.52	−0.20
TRN	−0.13	−0.31	0.06	−0.20	−0.38	−0.01	−0.25	−0.42	−0.08	−0.24	−0.40	−0.07
PAS	0.23	0.07	0.38	0.32	0.15	0.47	0.46	0.32	0.60	0.42	0.28	0.55
Subscale												
IA	−0.29	−0.45	−0.11	−0.35	−0.51	−0.17	−0.35	−0.52	−0.18	−0.41	−0.55	−0.26
IB	−0.08	−0.25	0.10	−0.16	−0.31	0.02	−0.23	−0.41	−0.05	−0.20	−0.36	−0.01
IM	−0.21	−0.38	−0.02	−0.26	−0.42	−0.07	−0.26	−0.43	−0.08	−0.30	−0.45	−0.12
IS	−0.24	−0.43	−0.04	−0.29	−0.47	−0.11	−0.35	−0.50	−0.18	−0.36	−0.52	−0.21
IC	−0.16	−0.34	0.02	−0.19	−0.36	−0.01	−0.23	−0.39	−0.05	−0.24	−0.38	−0.06
CR	−0.18	−0.33	−0.01	−0.25	−0.42	−0.07	−0.33	−0.49	−0.15	−0.31	−0.46	−0.16
MBEA	−0.03	−0.22	0.16	−0.07	−0.26	0.11	−0.09	−0.27	0.08	−0.09	−0.26	0.08
MBEP	0.23	0.05	0.39	0.33	0.17	0.48	0.40	0.24	0.55	0.39	0.26	0.53
LF	0.21	0.04	0.36	0.27	0.10	0.44	0.47	0.33	0.60	0.39	0.25	0.52
Outcome												
EE	−0.13	−0.29	0.05	−0.08	−0.26	0.11	−0.19	−0.35	−0.01	−0.16	−0.31	0.01
EFF	−0.28	−0.43	−0.10	−0.33	−0.48	−0.16	−0.39	−0.54	−0.23	−0.41	−0.54	−0.27
SAT	−0.29	−0.46	−0.10	−0.41	−0.57	−0.23	−0.37	−0.54	−0.20	−0.44	−0.59	−0.29

Note: Shaded cells denote statistical significance (light grey *p* < 0.05; dark grey *p* < 0.01); LB and UB refer to bootstrap 95% BCa CI lower and upper bounds, respectively.

**Table 11:** Pearson correlations (*r*) between personal, work-related, colleague-related, and overall burnout with demographic and other participants’ characteristics (*N* = 107).

Participant Charecteristic	Copenhagen Burnout (CBI)											
	Personal			Work-Related			Colleague-Related			Overall		
	*r*	LB	UB	*r*	LB	UB	*r*	LB	UB	*r*	LB	UB
Gender	0.30	0.11	0.47	0.21	0.03	0.40	−0.02	−0.20	0.17	0.19	−0.01	0.39
Age	0.09	−0.13	0.30	−0.09	−0.27	0.09	−0.01	−0.18	0.14	−0.02	−0.22	0.18
Marital status	−0.03	−0.22	0.14	0.06	−0.12	0.24	−0.07	−0.25	0.11	−0.01	−0.20	0.17
Specialty	0.09	−0.09	0.26	0.05	−0.13	0.23	0.15	−0.04	0.34	0.12	−0.07	0.29
Degree	−0.07	−0.27	0.13	−0.04	−0.23	0.17	−0.02	−0.20	0.17	−0.05	−0.25	0.16
Work Type	−0.23	−0.40	−0.05	−0.18	−0.36	0.01	−0.02	−0.21	0.18	−0.17	−0.35	0.02
Tenure	0.14	−0.06	0.32	0.06	−0.12	0.24	0.09	−0.09	0.25	0.11	−0.07	0.28

Note: Shaded cells denote statistical significance (light grey *p* < 0.05; dark grey *p* < 0.01); LB and UB refer to bootstrap 95% BCa CI lower and upper bounds, respectively. Gender (1 = male, 0 = female); Marital status (1 = married, 0 = not married); Specialty (1 = physician, 0 = other); Work type (1 = permanent, 0 = non-permanent). Tenure: 1 = (0–5], 2 = (5–10], 3 = (10–15], 4 = (15–20], 5 = (20+).

**Table 12:** Pearson correlations (*r*) between MLQ-5X leadership styles and outcomes with demographic and other participants’ characteristics (*N* = 107).

Perticipant Characteristic	MLQ Leadership Styles and Outcomes																	
	TRF			TRN			PAS			EE			EFF			SAT		
	*r*	LB	UB	*r*	LB	UB	*r*	LB	UB	*r*	LB	UB	*r*	LB	UB	*r*	LB	UB
Gender	0.00	−0.17	0.18	0.07	−0.12	0.26	0.05	−0.14	0.21	0.10	−0.07	0.28	−0.01	−0.18	0.18	−0.03	−0.20	0.16
Age	0.03	−0.17	0.23	0.06	−0.15	0.27	−0.08	−0.25	0.09	−0.14	−0.31	0.05	−0.04	−0.23	0.17	0.04	−0.15	0.24
Marital status	−0.21	−0.37	−0.02	−0.24	−0.42	−0.07	0.18	0.00	0.37	−0.07	−0.26	0.10	−0.19	−0.38	0.01	−0.12	−0.31	0.07
Specialty	0.19	−0.01	0.37	0.13	−0.07	0.33	−0.08	−0.26	0.12	0.17	−0.04	0.37	0.10	−0.10	0.31	0.11	−0.08	0.31
Degree	−0.15	−0.33	0.03	−0.18	−0.37	0.03	−0.03	−0.20	0.15	−0.10	−0.28	0.06	−0.04	−0.22	0.13	−0.01	−0.19	0.16
Work Type	0.11	−0.08	0.29	0.04	−0.14	0.22	−0.02	−0.22	0.18	0.19	0.00	0.36	0.04	−0.15	0.24	0.10	−0.11	0.29
Tenure	−0.14	−0.32	0.05	−0.10	−0.30	0.10	0.10	−0.07	0.27	−0.28	−0.44	−0.10	−0.11	−0.29	0.09	−0.08	−0.24	0.10

Note: Shaded cells denote statistical significance (light grey *p* < 0.05; dark grey *p* < 0.01); LB and UB refer to bootstrap 95% BCa CI lower and upper bounds, respectively. Gender (1 = male, 0 = female); Marital status (1 = married, 0 = not married); Specialty (1 = physician, 0 = other); Work type (1 = permanent, 0 = non-permanent). Tenure: 1 = (0–5], 2 = (5–10], 3 = (10–15], 4 = (15–20], 5 = (20+).

**Table 13:** Hierarchical Multiple Linear Regression Analysis for Dependent Variables: Personal Burnout (PB) and Work-related Burnout (WB).

Model	Predictor Variables	B	SE	β	t	*p*	95% CI for B		TOL	VIF
							LB	UB		
PB-1	Summary: R^2^ = 0.14, adj. R^2^ = 0.11, SE = 16.66, ΔR^2^ = 0.14, ΔF(3, 103) = 5.51, *p* < 0.001									
	(Constant)	48.54	3.30		14.71	0.00	41.99	55.08		
	Gender	10.47	3.53	0.29	2.97	0.00	3.47	17.46	0.90	1.11
	Specialty	0.48	3.42	0.01	0.14	0.89	−6.29	7.26	0.90	1.11
	Employment type	−7.66	3.25	−0.22	−2.36	0.02	−14.11	−1.22	0.99	1.01
PB-2	Summary: R^2^ = 0.20, adj. R^2^ = 0.15, SE = 16.29, ΔR^2^ = 0.06, ΔF(3, 100) = 2.55, *p =* 0.06, Durbin-Watson = 2.00									
	(Constant)	52.26	9.27		5.64	0.00	33.87	70.65		
	Gender	9.57	3.48	0.26	2.75	0.01	2.66	16.49	0.88	1.13
	Specialty	2.14	3.42	0.06	0.63	0.53	−4.63	8.92	0.86	1.16
	Employment type	−6.89	3.22	−0.19	−2.14	0.03	−13.27	−0.51	0.97	1.03
	Transformational	−5.49	4.28	−0.23	−1.28	0.20	−13.98	3.00	0.24	4.14
	Transactional	2.45	4.08	0.09	0.60	0.55	−5.64	10.55	0.34	2.95
	Passive/Avoidant	2.20	2.43	0.11	0.90	0.37	−2.63	7.03	0.55	1.80
WB-1	Summary: R^2^ = 0.07, adj. R^2^ = 0.05, SE = 20.19, ΔR^2^ = 0.07, ΔF(3, 103) = 2.74, *p =* 0.05									
	(Constant)	44.09	4.00		11.03	0.00	36.16	52.02		
	Gender	8.83	4.27	0.21	2.07	0.04	0.35	17.31	0.90	1.11
	Specialty	−0.20	4.14	0.00	−0.05	0.96	−8.41	8.01	0.90	1.11
	Employment type	−6.90	3.94	−0.17	−1.75	0.08	−14.71	0.91	0.99	1.01
WB-2	Summary: R^2^ = 0.18, adj. R^2^ = 0.13, SE = 19.25, ΔR^2^ = 0.11, ΔF(3, 100) = 4.43, *p =* 0.01, Durbin-Watson = 1.90									
	(Constant)	47.21	10.95		4.31	0.00	25.48	68.94		
	Gender	7.62	4.12	0.18	1.85	0.07	−0.55	15.79	0.88	1.13
	Specialty	2.13	4.04	0.05	0.53	0.60	−5.88	10.13	0.86	1.16
	Employment type	−6.00	3.80	−0.14	−1.58	0.12	−13.54	1.54	0.97	1.03
	Transformational	−5.66	5.06	−0.21	−1.12	0.27	−15.69	4.37	0.24	4.14
	Transactional	1.42	4.82	0.05	0.30	0.77	−8.14	10.99	0.34	2.95
	Passive/Avoidant	4.65	2.88	0.20	1.62	0.11	−1.06	10.35	0.55	1.80

Note: B = unstandardized coefficient; SE = standard error; β = standardized coefficient; LB/UB = lower/upper bounds of the 95% confidence interval (CI) for B; TOL = tolerance; VIF = variance inflation factor. Coding of binary predictors: gender (female = 1, male = 0), specialty (other = 1, doctor = 0), employment type (non-permanent = 1, permanent = 0).

**Table 14:** Hierarchical Multiple Linear Regression Analysis for Dependent Variables: Colleague-related Burnout (CB) and Overall-related Burnout (OB).

Model	Predictor Variables	B	SE	β	t	*p*	95% CI for B		TOL	VIF
							LB	UB		
CB-1	Summary: R^2^ = 0.03, adj. R^2^ = 0.00, SE = 22.49, ΔR^2^ = 0.03, ΔF(3, 103) = 1.04, *p =* 0.38									
	(Constant)	29.31	4.45		6.58	0.00	20.48	38.14		
	Gender	−3.39	4.76	−0.07	−0.71	0.48	−12.84	6.05	0.90	1.11
	Specialty	8.04	4.61	0.18	1.74	0.08	−1.11	17.19	0.90	1.11
	Employment type	−1.39	4.39	−0.03	−0.32	0.75	−10.09	7.31	0.99	1.01
CB-2	Summary: R^2^ = 0.27, adj. R^2^ = 0.22, SE = 19.81, ΔR^2^ = 0.24, ΔF(3, 100) = 10.89, *p <* 0.001, Durbin-Watson = 2.01									
	(Constant)	23.21	11.27		2.06	0.04	0.85	45.58		
	Gender	−5.28	4.24	−0.11	−1.25	0.22	−13.68	3.13	0.88	1.13
	Specialty	11.12	4.15	0.25	2.68	0.01	2.88	19.36	0.86	1.16
	Employment type	−0.53	3.91	−0.01	−0.14	0.89	−8.29	7.23	0.97	1.03
	Transformational	−4.47	5.20	−0.15	−0.86	0.39	−14.80	5.85	0.24	4.14
	Transactional	1.07	4.96	0.03	0.22	0.83	−8.78	10.92	0.34	2.95
	Passive/Avoidant	10.36	2.96	0.40	3.50	0.00	4.49	16.23	0.55	1.80
OB-1	Summary: R^2^ = 0.07, adj. R^2^ = 0.04, SE = 16.41, ΔR^2^ = 0.07, ΔF(3, 103) = 2.46, *p =* 0.07									
	(Constant)	40.83	3.25		12.56	0.00	34.38	47.27		
	Gender	5.49	3.47	0.16	1.58	0.12	−1.40	12.38	0.90	1.11
	Specialty	2.62	3.37	0.08	0.78	0.44	−4.06	9.29	0.90	1.11
	Employment type	−5.40	3.20	−0.16	−1.69	0.09	−11.75	0.95	0.99	1.01
OB-2	Summary: R^2^ = 0.26, adj. R^2^ = 0.21, SE = 14.86, ΔR^2^ = 0.19, ΔF(3, 100) = 8.52, *p <* 0.001, Durbin-Watson = 1.85									
	(Constant)	41.23	8.45		4.88	0.00	24.45	58.00		
	Gender	4.16	3.18	0.12	1.31	0.19	−2.14	10.47	0.88	1.13
	Specialty	4.97	3.11	0.15	1.60	0.11	−1.21	11.15	0.86	1.16
	Employment type	−4.55	2.93	−0.14	−1.55	0.12	−10.38	1.27	0.97	1.03
	Transformational	−5.23	3.90	−0.24	−1.34	0.18	−12.97	2.51	0.24	4.14
	Transactional	1.64	3.72	0.07	0.44	0.66	−5.75	9.02	0.34	2.95
	Passive/Avoidant	5.68	2.22	0.30	2.56	0.01	1.27	10.08	0.55	1.80

Note: B = unstandardized coefficient; SE = standard error; β = standardized coefficient; LB/UB = lower/upper bounds of the 95% confidence interval (CI) for B; TOL = tolerance; VIF = variance inflation factor. Coding of binary predictors: gender (female = 1, male = 0), specialty (other = 1, doctor = 0), employment type (non-permanent = 1, permanent = 0).

## Data Availability

The raw data supporting this article’s conclusions will be made available by the authors upon reasonable request.
